# Digital Extension Interactive Voice Response (IVR) mLearning: Lessons Learnt From Uganda Pig Value Chain

**DOI:** 10.3389/fvets.2021.611263

**Published:** 2021-06-28

**Authors:** Michel Dione, Edwin Kangethe, Elizabeth Jane Poole, Nicholas Ndiwa, Emily Ouma, Iddo Dror

**Affiliations:** ^1^International Livestock Research Institute, Dakar, Senegal; ^2^International Livestock Research Institute, Nairobi, Kenya; ^3^International Livestock Research Institute, Kampala, Uganda; ^4^International Livestock Research Institute, Addis Ababa, Ethiopia

**Keywords:** interactive voice response, participatory training, biosecurity, pig, Uganda (Sub-Saharan Africa)

## Abstract

We assessed the effectiveness of Interactive Voice Response (IVR) technology in delivering biosecurity messages for the control of African swine fever (ASF) in Uganda using a randomized controlled trial (RCT) with 408 smallholder pig farmers. Our results show that IVR technology significantly improved knowledge of farmers who had not been exposed to training on biosecurity. Furthermore, it enhanced knowledge for farmers who had received face-to-face (f2f) training in biosecurity. This group of farmers recorded the highest knowledge gain following IVR training compared to farmers who did not receive f2f training. IVR technology was perceived by farmers as a new technology capable of transforming their lives because it is time efficient, has high potential for resource saving and flexibility. IVR also seems to be gender sensitive as it addresses some of the constraints women face in accessing conventional extension services such as time. IVR is an innovative way for delivery of advisory information to pig farmers. The scalability of IVR technology could further be explored and its feasibility assessed for wider use by the extension systems in Uganda and elsewhere.

## Background

Agricultural extension/advisory services in Uganda face many challenges due to lack of capacity of the government to support long-term interventions. This stems from the fact that the public services since structural adjustment in the 1980s have pulled out almost entirely from their leading role as extension service provider. Thus, most farmers in many areas are left fending for themselves. Development organizations have attempted to fill in the gaps but with very limited success, considering the temporary nature of their programs. Research has shown that farmers' exposure to information is a key driver influencing their adoption of technologies and best practices (1). Individual and group face-to-face (f2f) extension methods have been the standard ways to channel information to farmers. However, these approaches have their limitations such as high cost of delivery (2), insufficient funds for supporting public extension, limited involvement of rural farmers and populations, particularly women in extension processes, and lack of research and appropriate extension methods (3). This limits coverage of extension services, particularly across rural regions, and adapting technological packages to community-specific contexts (4). Given the situation, farmers require enough information and exposure to the latest approaches to make use of science and technology in the field of agriculture to increase productivity of livestock and crops. Over the last decade, largely due to the spread of mobile phone technology in rural areas, Information and Communication Technology (ICT) demonstrated the positive and significant impact they can have on economic development by improving the business environment in rural areas (1). In the present times of technological development, mobile technology particularly mobile phones has become the most important tool of communication which can be accessed by farmers for agriculture-related information and knowledge (5). In Uganda, access to mobile phones had increased from 0.13 to 25 million people between 2000 and 2018 (6). The increasing access and use of ICT tools by smallholder farmers provide an opportunity to improve communication, thus relaying critical information and knowledge to farmers in situations where resources, both financial and human, are limited. These ICT tools when properly applied in the context of the overall extension and advisory services system have the potential to address in a timely and effective manner the existing challenges being faced in the area of extension and advisory services by many livestock farmers in developing countries such as Uganda. These technologies hold the potential for reaching significant scale at a relatively low cost, so there is an interest to better understand this “scaling mechanism” so that it can benefit extension systems working on similar issues in the nexus of research to development.

It is against this backdrop that we have partnered with local district government to pilot test innovative ways through ICT to deliver information to smallholder pig farmers in Uganda. Our objective is not to replace conventional f2f extension methods, but to augment extension and advisory service programming through the integration of appropriate ICT tools. We chose Interactive Voice Response (IVR), as a potential ICT tool to deliver critical information to pig farmers in Uganda. IVR is a telephony system that interacts with callers, records information, and directs calls to an appropriate database of prerecorded information in voice form. An IVR system can accept telephone input through the touch-tone keypad selection and provide the appropriate response in the form of voice. This technology has been used in the area of human healthcare to provide opportunities to educate as well as to monitor individuals on their self-management behaviors (7). It has also been used in agricultural extension in India (8) and Tanzania (9) to improve its efficiency of prevalent services. The area of application of biosecurity for the control of African swine fever (ASF) in smallholder pig systems was identified as an interesting pilot case for Uganda. This is because timely provision of biosecurity information and knowledge to pig farmers is considered the most effective way for controlling ASF since there is no vaccine available in Sub-Saharan Africa so far. However, adoption of biosecurity measures is highly dependent on a farmer's knowledge about the best practices and their incentive to apply them adequately (10, 11).

Another reason why we chose biosecurity is the ongoing work since 2015, originally part of the CGIAR Research Program on Livestock & Fish, now part of the CGIAR Research Program on Livestock (hereafter Livestock CRP) in Uganda, which included a Randomized Controlled Trial (RCT) across several sites that looked at the effects of traditional extension activities such as participatory training (referred here as f2f training) on Knowledge Attitude and Practices (KAP) of pig farmers on biosecurity. Results showed that there was a significant effect of biosecurity training on gain in knowledge by pigs farmers in target sites (11). The RCT had a baseline, as well as two consecutive monitoring assessments of treatment and control groups with regard to the biosecurity aspects of ASF. By leveraging this ongoing work, we expected to come up with interesting insights on whether and how the addition of digital extension (IVR technology) can augment conventional f2f training. Specifically, the work built on existing research partnerships in Uganda and supplemented these with technical expertise, knowledge, and skills in converting learning materials and modules into digital format, notably IVR, so that semi-literate and semi-numerate livestock owners can improve their knowledge about pig husbandry and animal healthcare. The study addressed the following research question: does IVR technology enhance traditional training approach? Therefore, the objective of the study was to evaluate the effectiveness of IVR technology on farmer knowledge about biosecurity. The study also documented perceptions of pig farmers about digital extension and provided learning and experiences on the role of ICT in strengthening extension systems and their scalability potential in the context of smallholder pig systems in Sub-Saharan Africa.

## Materials and Methods

### Study Area

The study was carried out in Masaka district where an RCT to evaluate the participatory f2f training was being implemented. Masaka district is located in the central region and has the highest pig population density in Uganda (>50 heads/km^2^) (12). Pig farming is an important economic venture for smallholder farmers who often keep a small number of pigs for income generation. Masaka district was part of the “Smallholder Pig Value Chain Development Project” (SPVCD) in Uganda, which is a research for development program running since 2011 to improve pig value chains in the country (13). In each district, villages with high pig population density were identified from census data. Areas with the highest ASF outbreaks, based on records from respective district veterinary offices, were considered as a proxy for high ASF prevalence. Villages were randomly and equally allocated to treatment and control groups.

### Randomization

The design of the study allows for evaluation of the effects of f2f participatory training (P), the effects of IVR messaging (V), and the interaction of participatory training and IVR messaging, or the combined effect of training with IVR messaging. It followed a complete factorial design with participatory training (yes/no) and IVR messaging (yes/no) as the two factors. This provides four groups of individuals (Table 1; Supplementary Material 1):

**Table 1:** RCT arms and actual numbers of farms/households who have participated in the study.

**Village**	**Group 1**	**Group 2**	**Group 3**	**Group 4**	**Total**
	**No participatory training and no IVR messaging (P–V–)**	**Participatory training and no IVR messaging (P+V–)**	**No participatory training and IVR messaging (P–V+)**	**Participatory training and IVR messaging (P+V+)**	
Kanyaga	30	–	–	–	30
Luwerekera	26	–	–	–	26
Butego	26	–	–	–	26
Kirumba A	24	–	–	–	24
Lukindu	–	28	–	–	28
Butaano	–	24	–	–	24
Kalagala	–	21	–	–	21
Kiyimbwme	–	27	–	–	27
Minyinya proper	–	–	29	–	29
Zzimwe	–	–	29	–	29
Mwalo	–	–	19	–	19
Kamugombwa	–	–	25	–	25
Sserinya	–	–	–	27	27
Nkoma	–	–	–	24	24
Kikumba–Katwe	–	–	–	25	25
Kyabakuza	–	–	–	24	24
Total					408

The selection of subcounties carried out during the previous RCT and this study utilized the same subcounties. Villages for groups 2 (P+V–) and 4 (P+V+) also came from the previous study with selection of new villages for groups 1 (P–V–) and 3 (P–V+) following the same criteria as described above (11). Because of the need to utilize villages from previous study, the randomization of villages to group was only carried out for the IVR factor. This means that villages from the previous study were randomized to be V– or V+ (groups 2 and 4) and the new villages were also randomized to be V– or V+ (groups 1 and 3).

### Sample Size Calculation

Sample size calculations for the main effect of participatory training and IVR messaging used a two-sample binomial proportion comparison between the two pairs of groups (i.e., P+ vs. P– or V+ vs. V–) for the response indicator of farmer knowledge. Similar to the previous RCT, the calculation assumes a 30% difference in knowledge (35 vs. 65%) between these as being significant. For the interaction effect, the same calculation was used but for comparisons made between any two groups. Allocation of individuals to group was carried out at the village level to ensure no spillover between individuals across the different groups. Therefore, sample size calculations were adjusted for intra-cluster (village) correlation (ICC), assumed to be low for the IVR messaging as this technology is aimed directly at individuals, but for participatory training, we use the ICC obtained during the previous RCT (0.38). The sample size utilized here is the most conservative required, i.e., 30 households per village derived from the power calculation. However, some villages had sample sizes slightly <30 because some farmers withdrew from the study or did not show up during training. However, this situation did not affect the quality of the study (Supplementary Material 1).

### Description of Extension Technologies

#### Participatory Training

The process of participatory training was described elsewhere (11). Prior to the study, a training manual was developed by the project team (14). The content of the training was focused on transmission and spread of ASF as well as measures for its control and prevention. Emphasis was put on key biosecurity measures that could make a difference in the control of ASF such as pig confinement, farm visit restriction, management of sick animals, disposal of dead animals, processing of swill, disinfection, and outbreak reporting. The manual's content and the training approach were, respectively validated and tested with farmers and district veterinary extension personnel. The training of farmers was administered by extension staff from respective district veterinary offices to all consenting pig farmers in the villages that belonged to the treatment group. The extension officers were trained by the project team on how to administer the training. Farmers were split into groups of 20–30 people per training session which lasted ~4 h. The training course was made of five sessions: ASF causes, symptoms, and transmission (1 h); biosecurity measures at farm level (1 h); proper control of pig movements and reporting (30 min); on-farm practical demonstration of biosecurity measures (1 h); and training evaluation (30 min). Since the target of the training was to improve farmers' knowledge of biosecurity, we focused on knowledge and skill-based lessons.

Several delivery methods were used during the training including plenary brainstorming, small-group discussion, story storytelling and practical demonstration of cleaning and disinfecting a pigsty, construction of a footbath, hand washing and disinfection, use of protective wears, swill processing, and disposal of dead pigs. Various tools/aids were used to relay the messages including photos (of diseased pigs), posters, film clips, and drawings. Farmers who faced ASF outbreaks could share their experiences with others to stimulate discussions among participants who then reflected on the strength and weaknesses of the biosecurity measures they applied to control the disease.

#### Development and Implementation of the IVR mLearning

##### Content

We designed the training course on biosecurity measures for ASF disease based on training manuals used during the f2f participatory training sessions. To adapt this content into IVR audio files, we followed a two-step process. Firstly, the f2f training manuals were curated to create brief lesson paragraphs ensuring that each paragraph contained one or two key information points that we intended the farmer to take up; this was done to avoid overload to the farmer and limit the lesson to information that the farmer could immediately try on their farm. Secondly, using a performing arts team, we adapted this technical and “classroom” type of content into a drama series set in the local context and recorded in one of the highly used local languages, Luganda. The drama series approach was used to make the content interesting and relatable to the farmer with an objective of making it easier to be remembered and enhance the chances of finishing the 10-part lesson.

##### System Design of the IVR mLearning Training Prototype

Our IVR system was an in-house prototype consisting of hardware, software, and telecommunication infrastructure service to provide the connection. The system ran on a Centos operating system upon which we installed the telephony application *Asterisk IVR PBX* by Digium and developed a python script to run the commands. The system was installed in a standard personal computer (PC) with a peripheral component interconnect express (PCIe) slot to accommodate the digital telephone interface card (Digium TE122P PCI Card) for the E1 Connection that was used for the voice service. The fiber connection was provided by a local Telco MTN *via* fiber connection. This system has the capacity of handling up to 900 simultaneous calls, which was considerably higher than our target group of 240 participants. The staff support required for creating and maintaining the system included (1) a content specialist/epidemiologist to provide the content needed by farmers; (2) an ICT4D business analyst who understood the problem by the epidemiology team and designed the ICT solution that would best solve the problem; (3) an ICT technician to install the software and maintain the service; and finally (4) a performing artist to adapt the content into a voice-recorded drama series.

##### Training via IVR mLearning System

**Figure 1:**
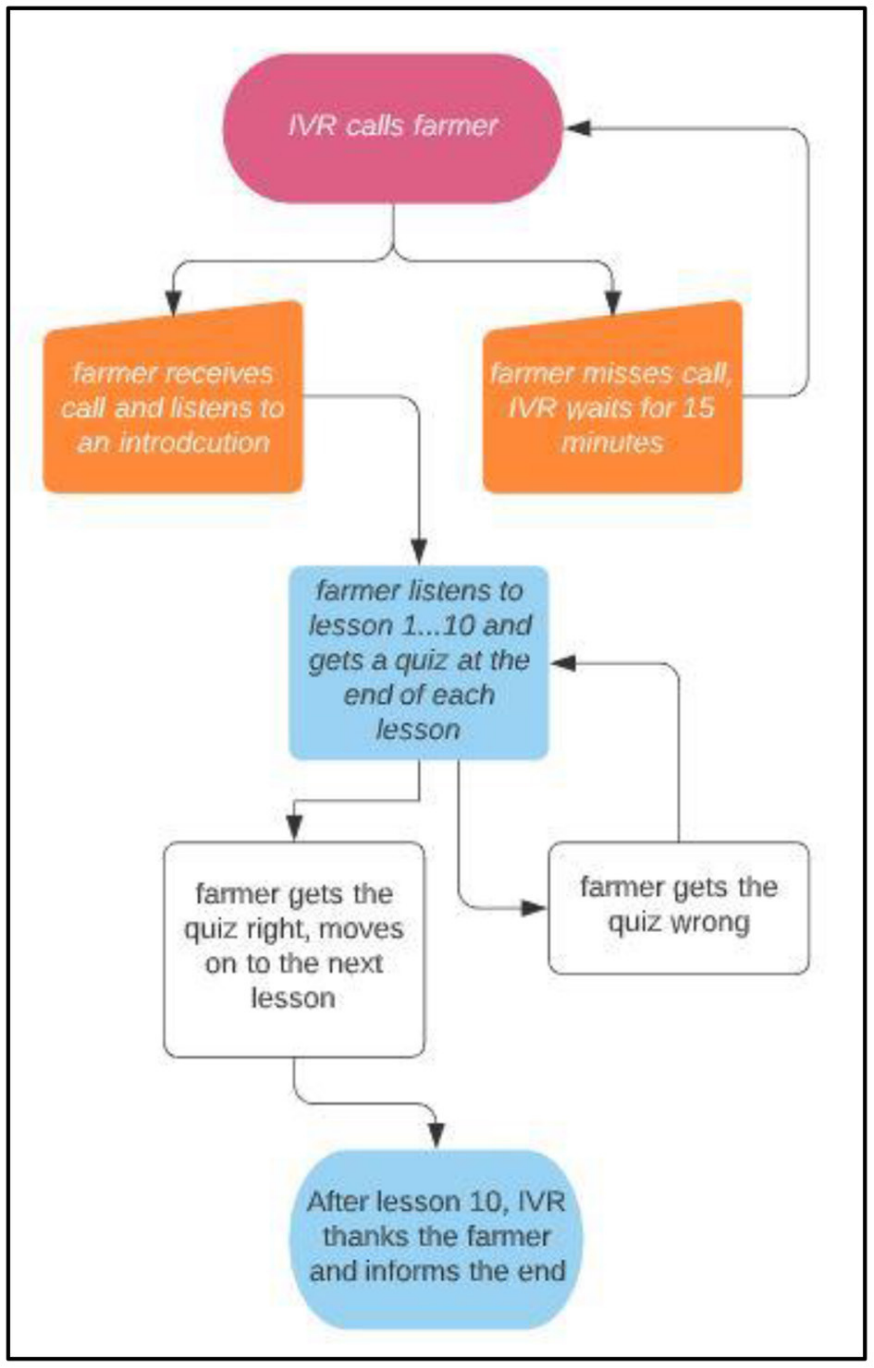
IVR processing diagram.

To raise awareness on the digital training course, we mobilized participants through the local government offices and extension workers, and the decision to participate was voluntary and formalized with a signed agreement. Participating farmers were then registered *via* their mobile phones and asked to indicate the time and day of the week they preferred to receive the IVR call. The course was designed to play two sessions a week unless the farmer opted to increase the occurrence of the sessions to a maximum of four a week to avoid information overload and possibly affect retention and adoption. The IVR system would then make the calls at the scheduled times. If a farmer needed to receive the call at any other time different to the registered time, they would call the system and the system would terminate the call and immediately return the call. The technology was only available to the registered farmers. Once the system called the farmer and the call was received, an introduction to the course and process was done followed by a lesson and a quiz. Depending on whether the farmer answered correctly or not, the system would determine whether they proceeded to the next lesson or would repeat the lesson once. The farmer would proceed this way until they completed the final lesson number 10 where they would be informed of the end of the course and thanked for their participation. After each call, the farmer had options to also repeat the lesson voluntarily, play the next lesson, or leave a message to the training team. A sample IVR flow is presented in Figure 1. The project assumed all costs and included free airtime as an incentive for answering post-lesson questions correctly, which was also a prerequisite for moving on to the next lesson (Figure 1).

#### Evaluation of the Interventions

The f2f participatory training began in April 2015 and lasted for 1 year (until May 2016), while the IVR technology which lasted 6 months started with a pilot on May 16, 2018, and ended on November 19, 2018, followed by an assessment (using the same/similar assessment template/indicators as the original project, for comparability). A few questions were added to the standard assessment tool to capture perceptions of farmers toward the technology and document lessons learnt. Prior to applying the technology, a baseline survey similar to the one carried out during the previous project was conducted to assess the level of knowledge of farmers about biosecurity and ASF control in all four RCT arms. Three months after administration of the IVR technology, the same survey was repeated to evaluate changes (knowledge gain by farmers) made by the training. In addition, 120 of the farmers who received the IVR training responded to the question that aimed at assessing their perception about the technology and document challenges they faced during the training.

#### Data Collection and Analysis

Field data collection and processing was carried using CSPro whereby initial data cleaning and validation was done. The cleaned data was then exported to STATA 16 for advanced data processing and analysis. The five-Likert scale system used during field data capture was recoded into a binary format (1, 0) whereby a value of 1 represented a scientifically correct response while 0 represented the wrong or undesired response. The recoding was necessary to adjust to the unidimensional scale, which is a fundamental assumption of the Item Response Theory (IRT) model, procedures for analyzing and obtaining information about the respondents, the questions asked (items), and the latent variable measuring the level of biosecurity practices among interviewed farmers. Using IRT, items that correctly captured the latent variable based on the discriminatory powers were retained in the model while those that did not were excluded. The retained items (questions) were used to fit a two-parameter logistic (2PL) IRT model to generate item characteristics curves (ICC), and item information functions. In the 2PL, the respondent's choice of the correct or wrong answer is dependent on the respondent's ability (knowledge) level, the item difficulty, and its discrimination. Item discrimination is the degree to which an item differentiates individuals with high knowledge level from individuals with low knowledge level, while an item's difficulty reflects the knowledge or level required for a respondent to have a 50% chance of answering the question (item) correctly. The individual respondent's overall knowledge (latent trait—designated theta) was estimated using an empirical Bayes estimator. Change in knowledge calculated as the difference between “after” training and “before” theta scores was then analyzed using a mixed-effects linear model with village as the random effect.

## Results

### Demographic Characteristics of the Participants

A total of 408 households participated in the pre- and post-training surveys, in four different groups. Group 1 (P–V–) had 99 participants, group 2 (P+V–) had 100 participants, group 3 (P–V+) had 102 participants, and group 4 (P+V+) had 107 participants. Although most of the sampled households (76%) were male headed, 58% of the respondents were female (Table 2).

**Table 2:** Household demographic characteristics (%).

**Characteristics**	**Variable**	**P+V+ (*n* = 107)**	**P+V– (*n* = 100)**	**P–V+ (*n* = 102)**	**P–V– (*n* = 99)**
Sex of household	Male	66	72	67	66
	Female	34	28	33	34
Sex of respondent	Male	28	45	24	34
	Female	72	55	76	66
Highest education level	No education	3	6	2	2
	Primary	49	50	60	69
	Secondary	35	29	33	26
	Post-secondary	12	15	5	3
Respondent role in the farm	Daily management	98	99	99	96
	Marketing	2	1	1	4
Number of respondents	99	100	102	107	

**Figure 2:**
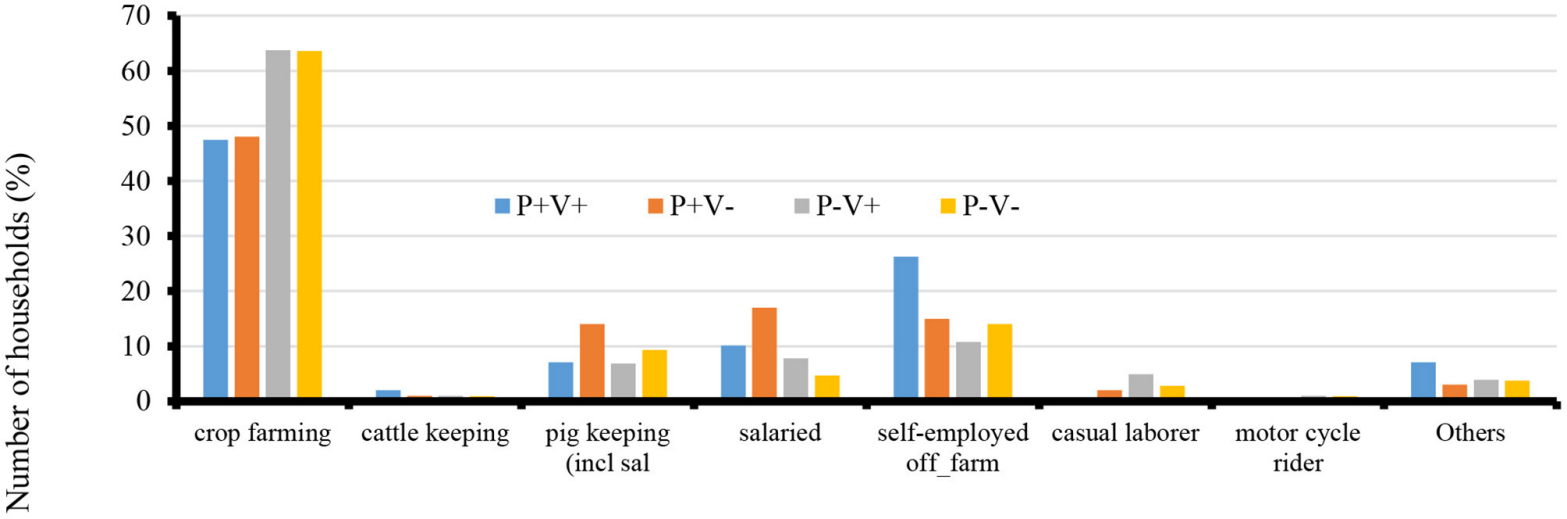
Main household income sources for the farmers.

The average age for respondents in both the treatment and control groups was 47 years with standard deviation of 14. Crop, pig farming, self-employed off farm, and salaried were the main sources of income for households in all groups (Figure 2). Overall, 14% of the respondents belonged to farmer groups with more respondents from the P+V+ group (27%) belonging to farmer groups, followed by P–V+ group (13%). P+V– and P–V– were all at 8%.

### Impacts of Training on Farmers' Knowledge

**Figure 3:**
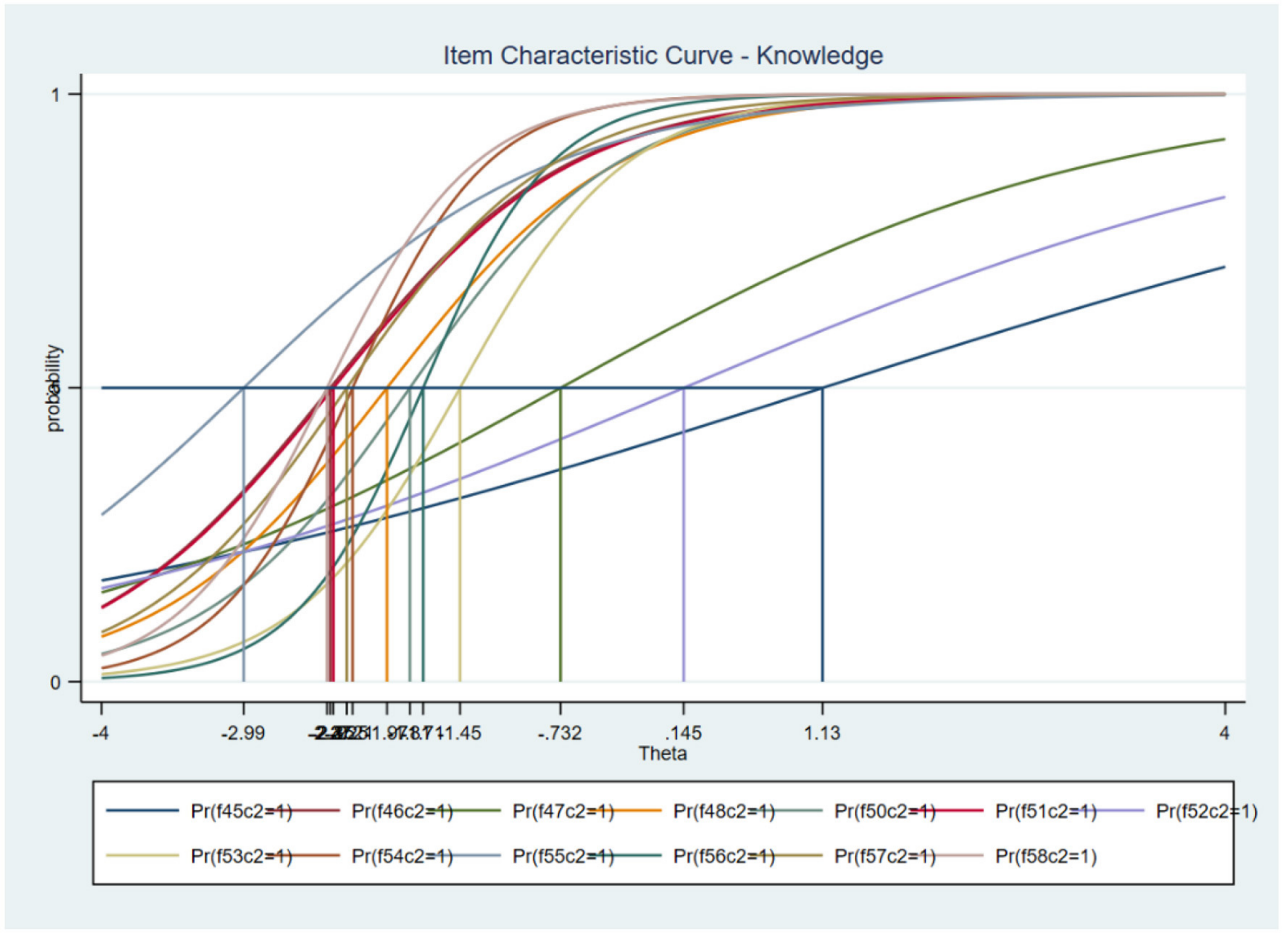
Item characteristic curve.

The percentage of P+V+ respondents correctly answering biosecurity questions consistently increased between phase 1 and phase 2 compared with other groups particularly P–V– group, which showed inconsistency (Appendix 1). Each biosecurity question was assessed for consistency using the IRT method, and their discriminatory and difficulty coefficients were generated (Appendix 2). The question on whether housed pigs “catch” ASF or not was the most difficult question with a coefficient of 1.13 and overall percentage of households correctly responding to it being less than 50% in both phases. This question was also the least discriminating question (discrimination coefficient = 0.31). The most discriminating question was the one of whether disinfecting farm tools controlled the spread of the disease or not which had a coefficient of 2.21. Figure 3 is the graphical representation of item difficulty and discrimination coefficients for each biosecurity question.

Individual knowledge gain was calculated as the change in an individual household's knowledge scores between phase 1 and phase 2 (Table 3). A positive knowledge gain showed an increase in knowledge after intervention (training) while a negative knowledge gain showed a drop in knowledge. Use of participatory methods showed a higher knowledge gain than other methods; use of IVR showed a smaller knowledge gain compared to non-IVR for both the trained and non-trained groups.

**Table 3:** Average knowledge gain by group.

**Group**	**Mean knowledge gain**	**Std. dev**.	**N**
P+V+	0.82	0.89	99
P+V–	0.77	0.96	100
P–V+	0.44	0.88	102
P–V–	0.30	0.84	107
Total	0.58	0.92	408

**Figure 4:**
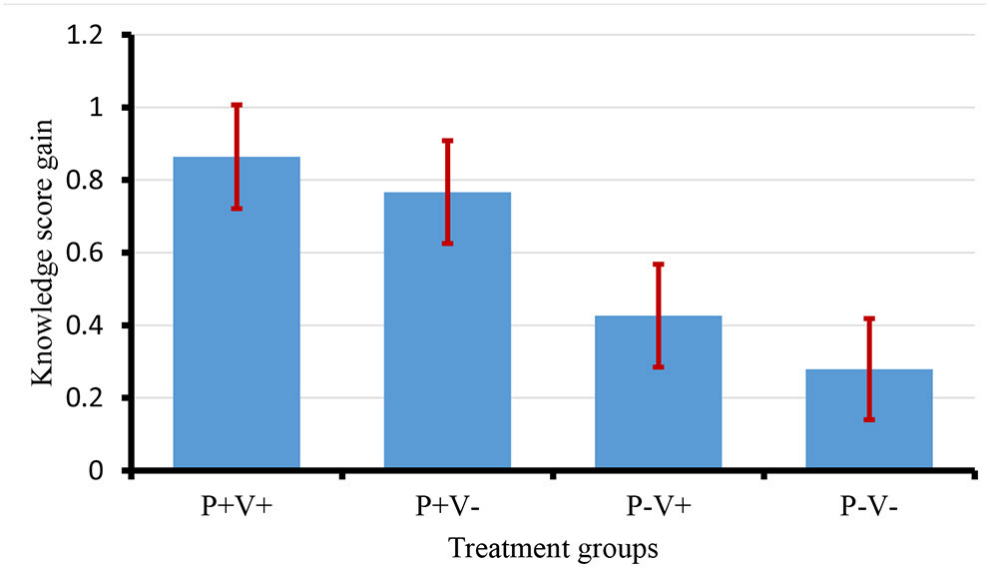
Adjusted knowledge change scores by group (standard error bars shown).

Figure 4 shows the least-square mean knowledge changes; the group who received both participatory training and IVR (P+V+) recorded a significantly higher change in knowledge scores than groups that did not receive participatory training (P–V+ *p* = 0.030, P–V– *p* = 0.003). There was also evidence that participatory training alone (P+V–) showed significantly higher gain in knowledge scores than no participatory training (P–V–) (*p* = 0.014). However, there was no significant difference in knowledge scores between the group receiving both participatory training and IVR (P+V+) and the group that received participatory training alone (P+V–) (*p* = 0.635) or between farmers receiving nothing (P–V–) and those receiving IVR only (P–V+) (*p* = 0.462).

A linear mixed model was used to test for differences between groups after adjusting for household characteristics and village as the random effect. Results from the linear mixed model are presented in Table 4. The only significant variables in the model are the group variable and farmer's years of experience. Farmers who had several years in farming experience showed a smaller increase in knowledge as compared to newer farmers (*p* = 0.012).

**Table 4:** Parameter estimates and significant of variables influencing knowledge gain.

**Wald chi**^**2**^**(11) = 23.64, log restricted-likelihood = −507.58, Prob > chi**^**2**^ **= 0.0143**						
**Knowledge gain**	**Coef**.	**Std. err**.	**Z**	***P* > z**	**(95% conf. interval)**	
Group (P+V+)	0	–	–	–	–	–
P+V–	−0.096	0.203	−0.48	0.635	−0.494	0.301
P–V+	−0.438	0.202	−2.17	**0.030**	−0.833	−0.042
P–V–	−0.584	0.199	−2.94	**0.003**	−0.974	−0.194
Gender of farmer (male)	0	–	–	–	–	–
Female	0.147	0.139	0.11	0.916	−0.258	0.288
Education level (no education)	0	–	–	–	–	–
At least primary education	−0.021	0.094	−0.22	0.827	−0.204	0.163
Experience in pig keeping (in years)	−0.013	0.005	−2.53	**0.012**	−0.023	0.003
Belong to group (months)	0	–	–	–	–	–
Yes—belong to group	−0.087	0.136	−0.63	0.526	−0.354	0.181
Marital status (married monogamous)	0	–	–	–	–	–
Married polygamous	−0.175	0.196	−0.89	0.371	−0.560	0.209
Widow/widower	0.244	0.178	1.37	0.170	−0.104	0.592
Divorced/separated	0.169	0.210	0.80	0.421	−0.242	0.580
Single	−0.235	0.164	−1.44	0.151	−0.556	0.086
_cons	1.057	0.175	6.04	0.000	0.714	1.400

### Perception of Farmers About IVR Technology

#### IVR Access and Use

One hundred and twenty farmers participated in the evaluation of the IVR access. Ninety-one percent (91%) of farmers rated their experience with the IVR as good to very good. Half of the farmers had received at least three training sessions with 39% receiving 6–10 training during the pilot phase. Most farmers (88%) had used their own mobile phone, while the remaining used those of their spouse, child, or neighbor. Seventy-three percent of farmers think that the audio-recordings of the training sessions were of good quality, and almost all farmers said they would welcome back the training (Table 5).

**Table 5:** Responses of farmers to the evaluation survey.

**Category**	**Men**	**%**	**Women**	**%**	**Total**	**%**
**What is your overall experience of using mobile phones to complete on-the-farm training**						
Very good	14	42.4	30	34.5	44	36.7
Good	17	51.5	49	56.3	66	55.0
Bad	2	6.1	7	8.0	9	7.5
Very bad	0	0	1	1.1	1	0.8
Total	33	100	87	100	120	100.0
**How many refresher trainings have you had since the start of the pilot?**						
None	8	24.2	14	16.1	22	18.3
1–2 trainings	7	21.2	30	34.5	37	30.8
3–5 trainings	1	3.0	13	14.9	14	11.7
6–10 trainings	17	51.5	30	34.5	47	39.2
Total	33	100	87	100	120	100.0
**Whose mobile phone are you using for this service**						
Mine	32	97.0	74	85.1	106	88.3
Son/daughter	0	0.0	3	3.4	3	2.5
Spouse	1	3.0	9	10.3	10	8.3
Neighbor	0	0.0	1	1.1	1	0.8
Total	33	100	87	100	120	100.0
**How clear was the audio recording**						
Very well	10	30.3	23	26.4	33	27.5
Well	18	54.5	37	42.5	55	45.8
Adequately	2	6.1	18	20.7	20	16.7
Poorly/very poorly	3	9.1	9	10.3	12	10.0
Total	33	100	87	100	120	100.0
**Would you like to take another course on mobile phone**						
Yes	32	96.97	84	96.6	116	96.7
No	1	3.03	2	2.3	4	3.3
Total	33	100	87	100	120	100.0

#### The Process

**Figure 5:**
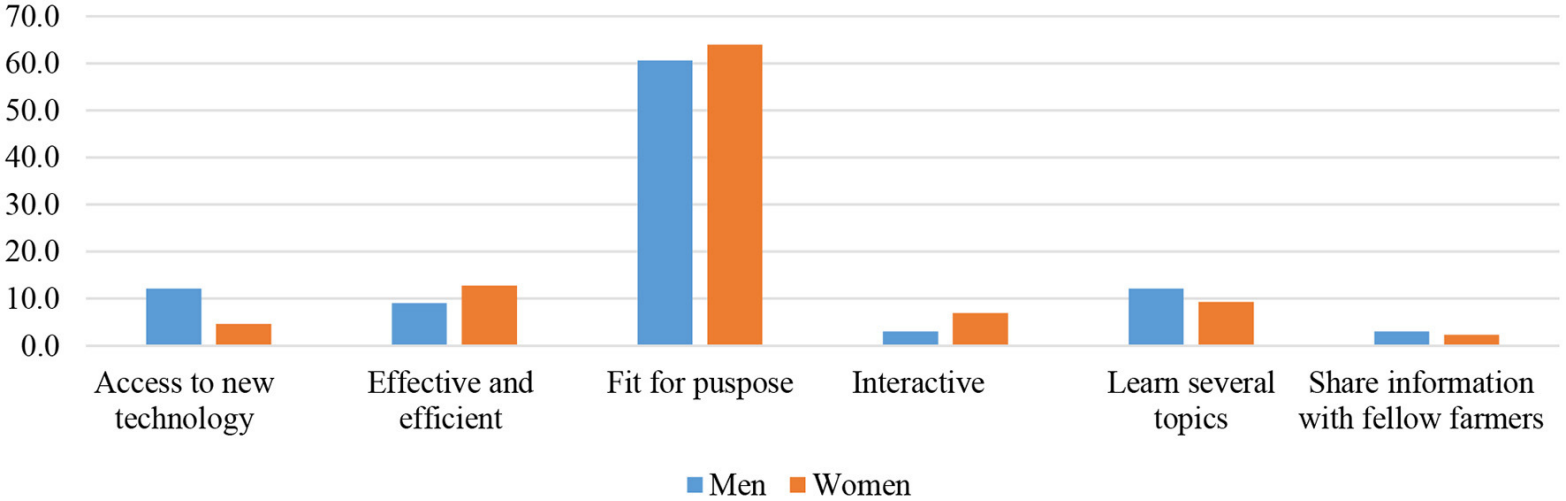
Why would you want more mobile trainings? (*n* = 119).

**Figure 6:**
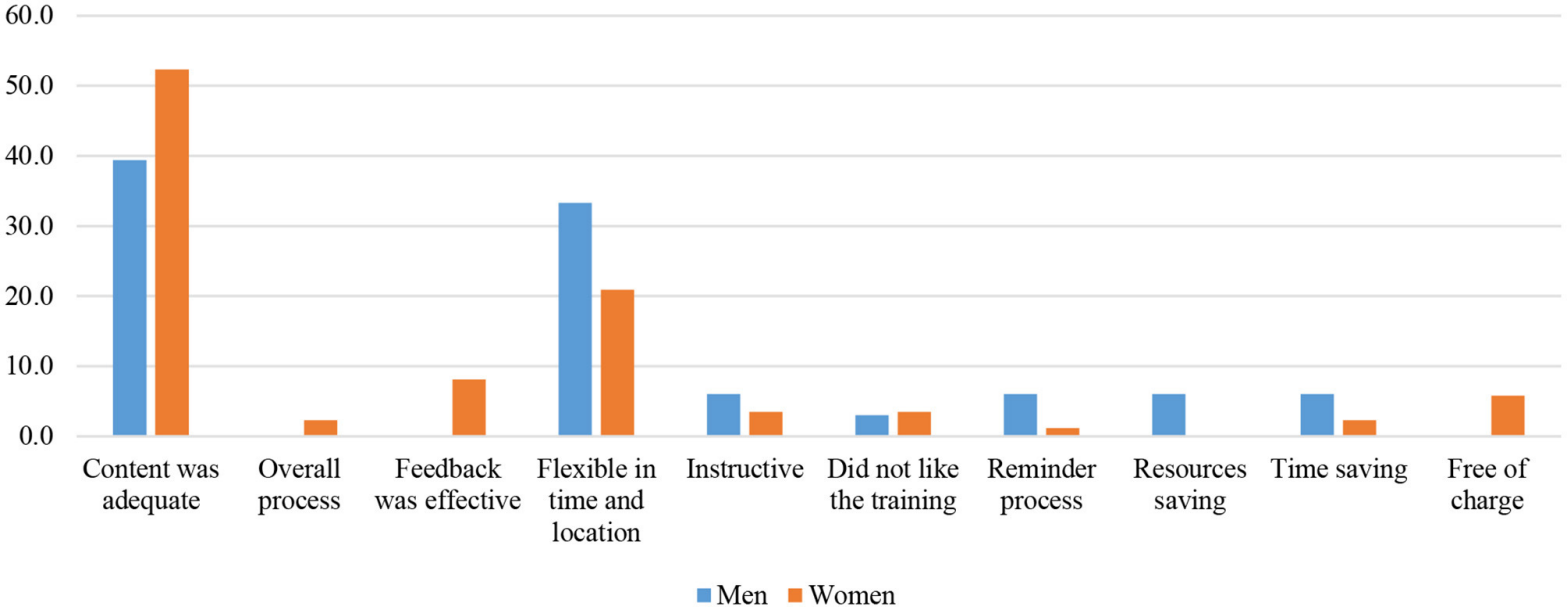
What in particular did you like about the mobile training? (*n* = 119).

Figures 5, 6 present the responses of the farmers to the evaluation question of the IVR technology. The majority of farmers believe that IVR is fit for purpose, meaning the course was mapped to their real needs since the content was adequate; hence, they were able to improve their knowledge about pig management and control of ASF. They also mentioned that training was efficient and effective in the sense that it was flexible in time since farmers could schedule their own session at their desired time of the day (especially after routine home work). Some quotes from farmers supported these statements:

“*It (the IVR) doesn't consume time like when farmers go for face-to-face trainings and at times the teachers don't appear yet with the mobile phone technology even if one is doing his or her work can just pause a bit to learn and resume to work*” *(women farmer from Masaka)*

For some farmers, it was important that farmers could log in and out to the training regardless of their physical position. This is illustrated with the following quote:

“*It is a lifelong learning anywhere you can learn” (women farmer)*

Most importantly, the IVR training was resource sensitive since farmers said they could save money through transport fees which they would have spent for f2f training. The training also enabled farmers to navigate across several sessions and hence decide which topic they are more interested to learn. The interactivity of the IVR was perceived as useful since automatic reminders could be sent to farmers about the topics to be covered in subsequent training sessions. The quote below denotes the importance of the feedback loop.

“*Trained in time and always reminded on previous topic before moving to next topic” (male farmer)*

**Figure 7:**
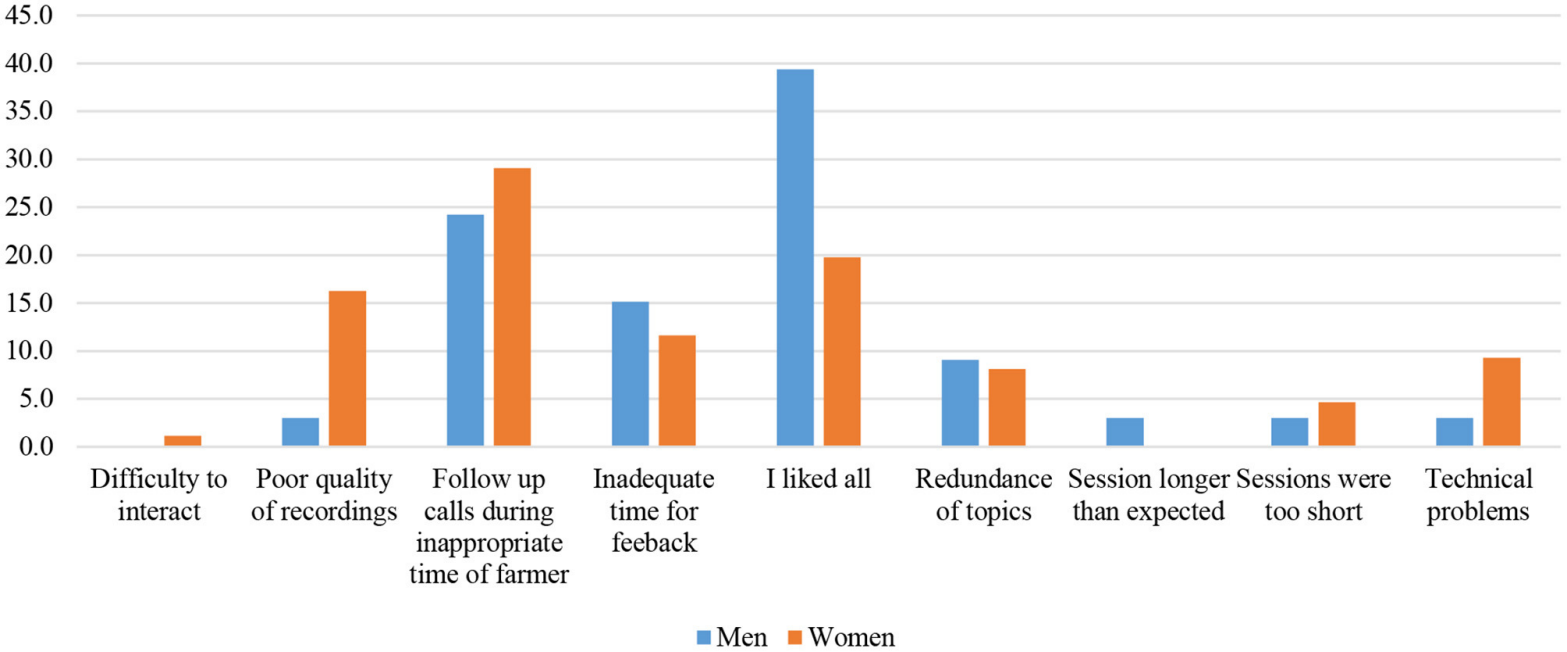
What didn't you like about the mobile training? (*n* = 119).

Among the drawbacks reported by the farmers (~30%), the follow-up calls were mainly mentioned to be happening during an inappropriate time of the day, especially when farmers are busy (Figure 7). Women complained more about this situation. Another point women complained about was the poor quality of the recording. The quotes below denote challenges reported by farmers. Some technical challenges occurred for some farmers mainly related to quality of the phone device and the availability of the network, both leading to poor quality of the tone.

“*The phone could go off, the language used was not clear and I could not understand and lastly i love to learn through the participatory way of teaching” (woman farmer)*

The f2f training was still seen as very important since trainers could interact longer with training participants and field demonstration could also be done easily. The quotes below show the limitation of the IVR training according to farmers.

“*The farmer understands better during face to face trainings” (woman farmer)*

“*The mobile phone training is not so friendly to me since I forget to connect” (woman farmer)*

“*It doesn't give time for one to think through what one can ask for thus a farmer failing to answer one's expectations” (woman farmer)*

### Technical Challenges Faced During Implementation of the IVR Technology

During the implementation of the IVR training, the researchers documented some technical challenges that could have contributed to the poor quality of the services highlighted by some farmers.

- Some farmers were not comfortable with the interactive nature of the IVR and often pressed the wrong phone device keys which lead to frustrations. Future deployments should minimize interactiveness as much as possible.
- Unreliable power source led to system downtimes that were impossible to pre-warn the farmers on. This can affect the perception of reliability of digital extension to farmers and affect future deployments. While future deployments might consider backup power generators, the associated higher costs would be better invested in going for a commercial system instead that would guarantee against this power problem.
- An in-house system in place of a commercial one suffered from recurrent lack of round-the-clock support to ensure the system stayed live; downtimes over the weekend and early evening were most affected as they would only be resolved at the earliest formal working day and hours. Adopting a commercial system would take care of this problem.
- The listening experience is also dependent on the quality of the mobile phone, and for some farmers, the phone was not audible enough.

## Discussion

In the context of agricultural development, information and communication technologies have played an important role in developing countries. ICTs are proving new approaches for communicating and sharing the information among livestock farmers (15, 16) to improve their knowledge and skills. Among modern ICTs, mobile phones serve as a means for effective transfer of knowledge and information about agricultural market and technology to farmers that enable them to apply the knowledge directly to improve their farming output and make easy access to market (17). Our study reveals that f2f training methods generated higher knowledge gain than other methods and use of IVR showed a smaller knowledge gain compared to non-IVR for both the trained and non-trained groups. However, a combination of both methodologies yields more knowledge gain. Elsewhere, ICT-based technologies have shown to be very effective in improving knowledge of farmers. That is the case of India where a study concluded that mobile agri-advisory service provided timely and relevant advice to farmers, and farmers adopted new practices based on information received through mobile services (3); the same was also seen in Tanzania (9). The use of mobile phones and emails had a positive impact on farm production of Chilean small farmers (18). However, conservative training (f2f training) still had a higher positive effect on knowledge gain of farmers in our study; this might be because farmers were still used to traditional training. Some farmers experienced technical challenges which limited them from completing the full course. One key thing to note is that the group that only received IVR had a knowledge score of 0.44. Given the short duration of the IVR intervention, there may still have been room to increase this if the intervention ran for a longer duration as the f2f. When combined (IVR + f2f training), the farmer knowledge gain was much higher. These results can be easily interpreted since the objective of the IVR technology was not to replace the conservative training f2f but to augment the delivery of knowledge and information and provide opportunities to farmers to adapt to new technologies that would give them more flexibility for self-learning. Farmer' experience in pig keeping had a negative and statistically significant coefficient in the model. An additional year of farming experienced was associated with a 0.01 reduction in knowledge score. This could probably imply that the knowledge levels of the more experienced farmers did not change much between baseline and endline surveys as they were already knowledgeable about biosecurity practices. f2f training enabled more interaction, but there was no opportunity to get hold of the trainer for further consultation after the training. This gap was filled by the IVR technology whereby the farmers had ample time to consult the services during their adequate time. The IVR system could not avail as much as possible of detailed information as it is the case of the f2f training. This is because the system device has a limit in capacity of information to process. However, there is possibility once the system is set up to add more technical content following farmer demand. While the IVR technology was dependent on power electricity and internet supply and human technical expertise, the f2f training depended on the capacity of the extension services to provide quality human resources to deliver the training, as well as financial capacity to support the field logistics including travels, communication, and meals of both trainers and trainees. Motivating farmers in adoption of new agricultural technologies remained a focal point of the agricultural extension (19). IVR was received by farmers as a new technology capable of transforming their lives since as they said it is time efficient, resource saving, and flexible to timing. ICT-based solutions were also viewed as an enabling tool for extension service delivery targeting poor rural farmers especially women (20). In our study, IVR technology seems to be gender sensitive—most farmers were able to use their own phone and plan the sessions the time they were more receptive without having to seek for approval from their partner, especially for women who are always overburdened with domestic chores and who follow the patriarchal settings of the communities. In this way, IVR technology has the potential to address some gender-related issues which would have raised following f2f trainings, whereby only household heads (most likely men in the study area) tend to attend trainings outside of the home (21). However, there is need for assessment cost development and implementation of both participatory and IVR technologies to better inform long-term investment in extension services by private and public health services.

Limitations of this study include possible spillover of information given that it was impossible to control information sharing among farmers between villages. All interviewers were sourced from the district veterinary office; hence, they are very socially close to the farmers. Therefore, bias associated with the nature of interviewers must also be considered. We expect that on some occasions, farmers gave misleading responses to hide their true perceptions. Farmers volunteered for treatment group, which could indicate a bias toward seeking additional training and knowledge and therefore more likely to increase participation in IVR training.

## Conclusion

IVR training improved the knowledge of farmers who have never been exposed to training on biosecurity before, and it also had a synergistic effect with f2f training by increasing the knowledge gain of farmers who had also been exposed to the conventional F2F trainings. The farmers who were exposed to trainings using both methods had the highest knowledge gain scores. IVR technology was perceived by farmers as a potential way for relaying information to farmers (time efficient, resource saving, and flexible). It seems to be sensitive since it enabled women to have space for planning their own training. Although IVR significantly increases knowledge of farmers about biosecurity, f2f training remains more effective. Delivery of the technology should be optimized in light of the challenges mentioned to make more positive impacts. IVR could be embedded in traditional extension systems to strengthen conventional training approaches and be used to channel important and urgent information for disease control such as biosecurity.

## Data Availability Statement

The raw data supporting the conclusions of this article will be made available by the authors, without undue reservation.

## Ethics Statement

The studies involving human participants were reviewed and approved by the Uganda National Committee for Scientific Technology with approval reference number A508 and by the Institutional Ethical Review Committee of College of Veterinary Medicine, Animal Resources and Biosecurity of Makerere University Uganda with approval reference SBLS.MD.2015. The patients/participants provided their written informed consent to participate in this study.

## Author Contributions

MD, EK, and ID conceived the study. MD developed the data collection tools. MD and EK collected the data and wrote the first draft of the manuscript. EJP and MD designed the RCT trial. NN and MD analyzed the data. EO and ID made critical review and edits to the final draft. All authors contributed to the article and approved the submitted version.

## Conflict of Interest

The authors declare that the research was conducted in the absence of any commercial or financial relationships that could be construed as a potential conflict of interest.

## Appendix

**Appendix 1:** Proportion of respondents correctly answering biosecurity knowledge assessment questions (P1—Phase 1, P2—Phase 2).

**Variable name**	**Variable description**	**P+V+**		**P+V–**		**P–V+**		**P–V–**		**Overall**	
		**P1**	**P2**	**P1**	**P2**	**P1**	**P2**	**P1**	**P2**	**P1**	**P2**
f45	Housed pigs may not catch ASF	40	47	34	33	40	48	51	41	41	42
f46	Footbath at the farm is a waste of money since it cannot prevent disease	76	97	80	96	92	99	93	96	86	97
f47	If I fence my house, the pigs will not catch the disease (ASF)	51	74	53	54	56	65	59	59	55	63
f48	My pigs can get sick when the traders get close to them	79	99	87	97	76	98	72	94	78	97
f49	My pigs can get sick when the vets get close to them without protective wears	46	37	54	45	43	39	49	41	48	41
f50	Birds or rodents can transmit the disease when they get in contact with the pigs	83	94	88	95	78	99	62	92	78	95
f51	If I isolate the newly coming pigs to my farm, I will stop the disease	79	97	77	96	93	97	94	94	86	96
f52	Pigs will catch the disease if the farm is clean	49	55	40	48	47	52	47	51	46	52
f53	If swill is heated before giving to pigs, chances of catching the disease is reduced	62	96	67	96	84	96	86	92	74	95
f54	Burying dead pigs reduces the disease spread	93	98	87	100	91	100	97	98	92	99
f55	Pigs will not get sick when they ingest offal's from infected dead pigs	83	95	89	96	89	97	88	98	87	97
f56	Un-disinfected farm tools can spread the disease	81	99	77	98	92	95	95	94	87	96
f57	Use of disinfectant is not good for the pigs	86	95	81	97	94	89	99	96	91	94
f58	I should avoid stray dogs from coming close to my pigs because they can transmit the disease to them	94	99	87	98	97	99	98	98	94	98
	Overall	72	85	71	82	77	84	78	82	75	83

**Appendix 2:** Difficulty and discrimination coefficients.

**Item**	**Discrim/diff**	**Coef**.	**Std. err**.	**z**	***P* > z**	**95% conf. interval**	
						**Lower**	**Upper**
f45	Discrim	0.31	0.16	1.92	0.06	–0.01	0.62
	Diff	1.13	0.62	1.83	0.07	–0.08	2.35
f46	Discrim	1.18	0.27	4.43	0.00	0.66	1.71
	Diff	–2.37	0.41	–5.85	0.00	–3.17	–1.58
f47	Discrim	0.53	0.18	2.93	0.00	0.17	0.88
	Diff	–0.73	0.28	–2.65	0.01	–1.27	–0.19
f48	Discrim	1.22	0.24	5.07	0.00	0.75	1.70
	Diff	–1.97	0.29	–6.85	0.00	–2.53	–1.40
f50	Discrim	1.37	0.29	4.66	0.00	0.79	1.94
	Diff	–1.81	0.26	–6.89	0.00	–2.32	–1.29
f51	Discrim	1.18	0.24	4.94	0.00	0.71	1.64
	Diff	–2.35	0.36	–6.59	0.00	–3.05	–1.65
f52	Discrim	0.40	0.14	2.81	0.01	0.12	0.68
	Diff	0.14	0.20	0.71	0.48	–0.26	0.54
f53	Discrim	1.70	0.30	5.69	0.00	1.12	2.29
	Diff	–1.45	0.16	–9.16	0.00	–1.76	–1.14
f54	Discrim	2.10	0.43	4.84	0.00	1.25	2.95
	Diff	–2.21	0.23	–9.44	0.00	–2.67	–1.75
f55	Discrim	0.91	0.23	3.96	0.00	0.46	1.37
	Diff	–2.99	0.62	–4.78	0.00	–4.21	–1.76
f56	Discrim	2.21	0.44	5.06	0.00	1.36	3.07
	Diff	–1.71	0.17	–10.00	0.00	–2.05	–1.38
f57	Discrim	1.37	0.29	4.77	0.00	0.81	1.93
	Diff	–2.25	0.34	–6.69	0.00	–2.91	–1.59
f58	Discrim	1.91	0.42	4.52	0.00	1.08	2.74
	Diff	–2.40	0.30	–8.03	0.00	–2.98	–1.81
