# Supplementary material for: Digital Extension Interactive Voice Response (IVR) mLearning: Lessons Learnt From Uganda Pig Value Chain
Source: Front Vet Sci. 2021 Jun 28;8:611263. doi: 10.3389/fvets.2021.611263 (PMC8273433; doi:10.3389/fvets.2021.611263)
Supplement: Supplementary file 1 [file Data_Sheet_1.docx]

Supplementary material 1: RCT arms and locations

Villages (all in different parishes) – Number of households shown next to name

**Group 1 – P-V-**

Kanyaga - 30

**Group 2 – P+V-**

Lukindu - 28

**Group 3 – P-V+**

Minyinya proper - 29

**Group 4 – P+V+**

Sserinya - 27

**Group 1 – P-V-**

Luwerekera - 26

**Group 2 – P+V-**

Butaano - 24

**Group 3 – P-V+**

Zzimwe - 29

**Group 4 – P+V+**

Nkoma - 24

**Group 1 – P-V-**

Butego - 26

**Group 2 – P+V-**

Kalagala - 21

**Group 3 – P-V+**

Mwalo – 19

**Group 4 – P+V+**

Kikumba-Katwe – 25

**Group 1 – P-V-**

Kirumba A - 24

**Group 2 – P+V-**

Kiyimbwme - 27

**Group 3 – P-V+**

Kamugombwa-25

**Group 4 – P+V+**

Kyabakuza – 24

**Kyanamukaka subcounty**

**Katwe-Butego sub-county**

**Masaka District (Central Uganda)**

Supplementary material 2: Sample size computation

| Response variable = Knowledge of biosecurity (% score) | Information obtained from previous study & estimate of 'significant' change that would be expected | | | Fixed by design | | V+ villages in study = 8 | Villages in study = 16 |
| --- | --- | --- | --- | --- | --- | --- | --- |
| **Effect to evaluate** (All calculations use 80% power at 5% level of significance) | **Baseline & Control % score** | **Post-intervention % score** | **Intra-cluster correlation (Village)** | **No. villages per 'Trt'** | **No. households per village to evaluate** | **No. households in IVR villages** | **Total number of households** |
| Biosecurity main effect | 50 | 70 | 0.01 | 8 | 11 | 88 | 176 |
| IVR main effect | 50 | 70 | 0.01 | 8 | 11 | 88 | 176 |
| Interaction of Biosecurity and IVR | | | | | | | |
| Training + IVR vs. No intervention | 50 | 80 | 0.01 | 4 | 10 | 80 | 160 |
| Training + IVR vs. Training OR IVR only | 70 | 85 | 0.01 | 4 | 30 | 240 | 480 |
